# Building Emergency Response Capacity: Multi-Career-Stage Social Workers’ Engagement with Homeless Sector during the First Two Waves of COVID-19 in Halifax, Nova Scotia, Canada

**DOI:** 10.3390/ijerph191912713

**Published:** 2022-10-05

**Authors:** Haorui Wu, Jeff Karabanow, Tonya Hoddinott

**Affiliations:** School of Social Work, Dalhousie University, Halifax, NS B3H 4R2, Canada

**Keywords:** COVID-19, emergency response interventions and challenges, critical reflection, persons experiencing homelessness, social work education

## Abstract

The dramatic increase of global extreme events (e.g., natural, technological, and willful hazards) propels social workers to be equipped with emergency response capacity, supporting affected individuals, families, and communities to prepare, respond, and recover from disasters. Although social workers have historically been engaged in emergency response, social work curriculum and professional training remain slow to adapt, jeopardizing their capacity to support the vulnerable and marginalized populations, who have always been disproportionately affected by extreme events. In response to this deficit, this article utilizes a critical reflection approach to examine three social workers’ (a senior faculty, a junior faculty, and a social work student) interventions and challenges in their emergency response to persons experiencing homelessness (PEHs) during the first two waves of COVID-19 in Halifax, Nova Scotia, Canada (March 2020 to March 2021). The cross-career-stage reflections and analyses exhibit these three social workers’ COVID-19-specific emergency response efforts: a top-down advocacy effort for social development and policy, a bottom-up cognitive effort to comprehend the community’s dynamics, and a disaster-driven self-care effort. These three types of effort demonstrate a greater need for social work education and professional training, to develop more disaster-specific components to contribute to building the emergency response capacity of the next generation of social workers through in-classroom pedagogical enhancement and on-site field education training, better supporting PEHs and other vulnerable and marginalized groups living in the diverse context of extreme events in Canada and internationally.

## 1. Introduction

The rapid global spread of the coronavirus has stimulated a systematic examination of international communities’ emergency response capacities, revealing that the operation of essential societal functioning (e.g., healthcare, critical goods, and infrastructure) relies on essential workers [1]. Although global citizens actively salute healthcare professionals in the traditional healthcare domains (e.g., hospitals and COVID-19 clinics), other essential healthcare professionals are often less appreciated, particularly social workers [2]. Social workers provide health and social services, often supporting the underrepresented and at-risk in our societies, including children, the elderly, persons experiencing homelessness (PEHs), and victims of domestic violence [3].

Despite experiencing risks in general practice (such as client violence, verbal abuse, and other assaults [4], and working amid the loss of lives, the jeopardization of human rights, and COVID-19 public health mitigations), social workers have continued to tirelessly advocate for vulnerable and marginalized populations [5]. Yet challenges associated with the current pandemic, including possible stigmatization [6] and escalated rates of domestic violence [7], continue to jeopardize the health and well-being of frontline social workers and their families, and even threaten their lives. Indeed, the current UK National Statistics reveal that the British national death rate among social care staff involving COVID-19 is at least twice that of healthcare workers [8]. Hence, the augmentation of social workers’ emergency response capacity is urgently needed. However, building this emergency response capacity among social workers so that they may effectively support individuals, families, and communities affected by extreme events, while maintaining their health and well-being, has been slow to develop in social work curriculum and professional training, or at least in the Canadian context of social work education [3].

The three authors—a senior faculty, a junior faculty, and an undergraduate student from the School of Social Work at Dalhousie University, Halifax, Canada—have collaboratively engaged in social work research, practice, education, and policymaking supporting PEHs in the Halifax Regional Municipality (HRM), the capital region of Nova Scotia (N.S.), Canada since the onset of COVID-19, in March 2020. In collaboration with the homeless sector, the three social work researchers and social work practitioners conducted rapid response research and developed practical interventions addressing the PEHs’ unique health, social, and economic challenges and informed related PEH-targeted social service programs and policies. The homeless sector consists of PEHs and service providers affiliated with public, private, and not-for-profit organizations, such as homeless shelters, health clinics, public health agencies, and different departments at various governmental levels. This collaboration has stimulated critical reflection and cross-career-stage analysis comparison, strengthening social work emergency response efforts by examining existing social work education and professional training. Through examining the three social workers’ individual and collective COVID-19-driven interventions and challenges with the homeless sector, this paper aims to shed light on identifying critical components in building social work-specific emergency response capacity that best serves vulnerable and marginalized populations, while maintaining the social workers’ health and well-being.

## 2. Social Workers in Disaster Settings

Social workers have a long history of engagement in disaster and emergency management scenarios, providing health and social services and acting as advocates for social and environmental justice for individuals disproportionately affected by extreme events [8]. The current global COVID-19 pandemic produces compounded impacts on the already disastrous status of PEHs and increases the demand for social work intervention to address PEHs’ unique challenges and support their pandemic recovery. Homelessness and COVID-19 jointly build a living platform on which social work professionals can develop their emergency response interventions.

### 2.1. Supporting Vulnerable and Marginalised Groups in Disaster Settings

The upward global tendency of extreme events (e.g., wildfires, epidemics, radiation exposures, and mass shootings) calls for social workers’ individual/family-driven and community-based expertise in providing rapid, relevant service and resource delivery to affected inhabitants, even as they may face more significant challenges across the disaster cycle [9]. In 1997, Dodds and Nuehring [10] developed the first research-practice conceptual framework to guide disaster-specific social work interventions at the micro (working with individuals and families), mezzo (engaging in communities and organizations), and macro (informing programs and social policies) levels. This framework highlighted the implementation of a qualitative approach to better understand the local residents’ unique needs in disaster settings and to inform related service programs and social policies in supporting the affected dwellers [10]. Yanay and Benjamin [11] further identified four areas of core expertise, that advance social workers’ coping capacities in complex disasters, including (i) understanding the characteristics at the individual, family, and community levels, (ii) emergency/crisis interventions, (iii) apprehending local social welfare systems and relevant social service programs, and (iv) networking capacities. While over three decades of development, disaster and emergency management components have existed in social work curriculum and professional training, significant theoretical, methodological, and practical gaps remain, causing delays in educating future social workers to respond to the rapidly escalating context of climate change, disasters, and other worldwide crises [12]. The current pandemic and its broad spectrum of catastrophic impacts on international communities reiterate the importance and urgency of improving the social work-specific emergency response capacity.

The rapidity of COVID-19 and related public health restrictions resulted in worldwide temporary closures of most community-based social services; however, pandemic-driven “pop-up” social service agencies, where frontline social workers served, have provided essential support for “left-behind” groups (e.g., PEHs, 2SLBGTQIA+, and women and children experiencing family violence and abuse) [13]. Other pandemic-associated factors, such as budget cuts and shortages of qualified employees, have negatively impacted these services and brought about tremendous “first-time” challenges for their operation. Some pop-up emergency shelters engaged a harm-reduction service approach [14], leaving unprepared and inexperienced frontline essential workers to seek support, training, and guidance while maintaining public health precautions. After the first shift at one of these pop-up homeless shelters, the social work student working there (co-author) noted, “it felt like it was slapped together, and no one was running it…” Most of these temporary agencies were designed to provide intensive service rather than offer new staff action-oriented training and related support. Thus, new frontline social workers, most of whom are recently graduated social workers and social work students, did not have enough tangible coping capacities. These inexperienced social workers’ challenges highlight the need to strengthen disaster and emergency response components in social work education to further build their emergency response capacity.

### 2.2. Social Worker Self-Maintenance in Disaster Settings

COVID-19 has highlighted the need for better self-maintenance of frontliners [15]. Public health social distancing measures increased the likelihood that social workers would confront high-risk situations alone, such as engaging with drug and alcohol-dependent PEHs [16]. Amidst the growing client violence, public health protocols limited new practitioners’ access to timely support from their senior colleagues and their organizations. Moreover, vulnerable clients generally have a much higher rate of asymptomatic or mild infection [17]; they can be ‘super-spreaders’ and re-infect their peers because of limited public health support [18]. This evidence triggered a high risk of exposure and transmission among frontline employees, worsened by the lack of organizational emergency planning and support. The temporary/junior nature of frontliners often led to last-minute attempts to ensure the safety of practitioners and clients, where wishful thinking was more common than actual safety procedures. Another factor was that the self-advocacy capacity of inexperienced frontliners was hobbled by fear of losing employment during a pandemic. The integration of all these factors highlighted the need to build a better emergency response capacity, in social work education and professional training, for the current as well as for future extreme events [19].

The dangerous nature of the social work profession has propelled social work educators, researchers, and practitioners worldwide to collectively strengthen future social work practitioners’ capacity to prevent professional risks, while maintaining a high quality of service [20,21]. Accordingly, post-secondary social work education has developed related courses and training modules to build students’ self-care and self-protection capacity. Field education directly engages students to learn client-oriented practice skills by pairing them with qualified senior practitioners. Most training focuses on non-disaster situations, making knowledge transfer and skills related to disaster and emergency scenarios imperative [3]. Although immediately after the outbreak, professional associations provided some self-protection-orientated practice recommendations and guidelines for COVID-19 (e.g., British Association of Social Work [22] and Canadian Association of Social Work [23]), however, the varying levels of emergency response experience, knowledge, and skills among social work practitioners may limit their ability to intensively build related capacity without suitable hands-on guidance.

### 2.3. COVID-19 and Homelessness

Homelessness, in and of itself, is a disaster, reflecting systemic inequalities associated with the full array of societal dimensions (e.g., physical, political, social, economic, and cultural) [24]. Furthermore, PEHs cover the entire demographic spectrum, for example, from youth to older adults [25,26], from men to women and other gender and sexual minorities [27,28], from immigrants to refugees [29], and from Indigenous to other ethnic minorities [30,31]. Understanding the complexity of homelessness requires a comprehensive approach in fathoming PEHs’ diverse vulnerabilities. Only then can the development of targeted practice and service programs be done, and related social policies be informed, and hopefully increase the number of social workers that are engaged in child protection, criminal justice, healthcare, and public policy.

In addition to providing general social and health care designated for PEHs, social work researchers and practitioners provide evidence-based strategies to support social policy improvement, such as poverty reduction [32], affordable housing [33], and reducing/eliminating social stigma and stereotype [34], addressing the fundamental and structural barriers that trigger homelessness in the first place. Currently, social work education supporting PEHs has primarily focused on homelessness rather than engaging this issue within the context of disaster and emergency. The global COVID-19 pandemic has surfaced the need to contextualize homelessness in disaster settings and mobilize the domestic evidence to the international scope.

COVID-19, like other extreme events, compounding with the previously disastrous condition of homelessness, has worsened the PEHs’ already vulnerable and marginalized status. Closures, during COVID-19, of community-based facilities deprived PEHs’ basic living rights, such as access to washrooms, food, and primary healthcare [35]. Furthermore, the prevalence of substance use, mental health challenges, and limited access to social services among PEHs triggered additional challenges for the overwhelmed healthcare system and related support workers (such as what occurred in the “pop-up” shelters) during the pandemic [36]. Additionally, supporting PEHs can negatively affect the social workers’ health and well-being even during non-disaster situations [37]. Indeed, Wirth and colleagues [38] discovered that German social workers in homeless aid show long-term psychological strain when lacking necessary workplace training, team support, and required health benefits. COVID-19 has complicated these existing challenges and added additional risk layers to already exhausted social workers and other support personnel. Since social workers are increasingly considered essential workers in the disaster and emergency management sector worldwide [39], social work research, education, and practice should contribute to building future social work professionals’ coping capacities toward compounded disaster impacts.

The profound societal influences of COVID-19 provide a valuable platform for research, learning, practice, and engaging social work professionals. The ongoing disaster of homelessness joined with the current COVID-19 has complicated the challenges for social work interventions, urgently calling for the building of social workers’ emergency response capacity. The full spectrum of societal vulnerabilities and demographic dimensions associated with PEHs indicate that the emergency response capacity may be transferable to support other vulnerable and marginalized groups during COVID-19 and future extreme events domestically and internationally. The paucity of emergency response components in the current social work education and professional training remains, especially in the Canadian social work education context [3]. Since social workers have been widely engaged in supporting PEHs during COVID-19, what core social work components could be generated from their pandemic-specific experience that aims to strengthen professional social work training?

## 3. Methods

Since the onset of the first wave of COVID-19 in HRM during March 2020, the three authors had collaborated with the homeless sector to develop different research and practice interventions to support the PEHs. The first wave of COVID-19 transformed the HRM, which is a medium-sized urban area and the most populated municipality on the Canadian east coast, as well as the economic, cultural, and transportation centers of the Atlantic Region of Canada, into a “dead zone”; namely, all non-essential service public spaces, including restaurants, libraries, and public washrooms, were closed, and all persons were requested to “stay the blazes home” [40]. Later, HRM became the epicenter of COVID-19 in Atlantic Canada, with the highest infection and death rate among the four Atlantic provinces [41]. Furthermore, the COVID-19 pandemic and its related societal influences doubled the number of chronically homeless in the HRM [42]. Shelter operators and other stakeholders advocated for a safer, healthier, and more dignified model for the city and province to house PEHs as guests in hotel rooms [35]. Later, this innovative model was widely accepted across Canada, North America, and beyond. These COVID-19 and PEH-specific environments encouraged the three authors’ critical reflections regarding their individual and collective interventions and challenges during the COVID-19 emergency response.

### 3.1. A Multi-Career-Stage Social Work Team

The COVID-19-driven research and practice environment in the HRM engaged the three authors, who are at different stages of their social work careers, contributing to PEH-specific COVID-19 quick response efforts. Their academic and demographic details are shown in Table 1 and detailed below:

*The senior faculty*, whose social work research and practice agenda predominately focuses on the homeless sector [43,44], participated in a COVID-19 Working Group, where different community-based service stakeholders (e.g., homeless shelters, public health agencies, and emergency management departments) working with PEHs throughout major metropolitan areas across North America (e.g., Boston, Massachusetts, and Halifax, N.S.) collaboratively explored best strategies from the field. He co-investigated two PEHs-specific COVID-19 quick response research projects with the junior faculty, qualitatively examining the health and social influences of COVID-19 on PEHs in N.S.

*The junior faculty*, an international hazards and disaster researcher and practitioner, primarily investigates vulnerable and marginalized groups’ diverse vulnerabilities in disaster settings. He is developing evidence-based interventions to build community resilience capacity. Building on the swiftly unfolding global pandemic, he has been integrating and strengthening the disaster and emergency management component into the core social work curriculum. In addition to the two Nova Scotian-based projects, he has led other projects exploring vulnerable populations’ COVID-19-driven vulnerabilities, including projects involving people with (dis)Abilities [45], immigrants and refugees, retail store employees, and PEHs.

*The social work undergraduate student* served as a research assistant in the junior faculty’s projects and also worked as a social work practitioner in two of the “pop-up” homeless shelters in the HRM during the COVID-19 epidemic, during the time she was completing the degree-required courses for her Bachelor of Social Work and finishing her field education. The challenges she faced practicing in these “pop-up” homeless shelters stimulated her re-examination of her social work education and professional training and was an impetus for the two faculty members to re-review their social work pedagogical interventions.

### 3.2. Critical Reflections

During the first two waves of COVID-19 in HRM, from March 2020 to March 2021, the three authors engaged in various aspects of social work-specific PEH-driven, community-target research, education, and practice initiatives in the HRM through grassroots clinical, policy, and other relevant approaches. They supported the development of the hotel transferring model, provided experimental research to support related policy decision-making, and directly provided service in homeless shelters. In addition to their research and practice roles, they were victims and survivors of the COVID-19 pandemic, client violence, and other pandemic-related situations. These experiences encouraged their ongoing reflections regarding their research and practice interventions associated with PEHs. They had bi-weekly or monthly virtual meetings and frequently communicated through email and phone to report their research progress, share their practice experience, and discuss how the lessons learned from the field would inform social work education and training.

These regular meetings and communication enabled a critical reflection platform, formulating the methodological strategy to conceptualize this manuscript. As a fundamental cognitive practice approach [46], critical reflection is the primary pedagogical approach in health/social care and related disciplines [47], such as social work [48], nursing [49], psychology [50], and education [51]. Particularly, critical reflection serves as a continuous learning and development tool, converting the researchers, practitioners, and educators’ individual and collective experiences into learning and, in turn, encourages them to ongoingly examine their research, practice, and education interventions [52]. Hence, critical reflection is precisely aligned with the research aim of this article because it has effectively assisted the three authors to examine their in-field interventions and challenges and apply these outcomes to improve their judgment, approaches, decisions, and interventions in support of PEHs in the COVID-19 emergence response. These field-based efforts illustrate the critical components in disaster-specific social work education, further shedding light on building social workers’ emergency response capacity.

**Figure 1:**
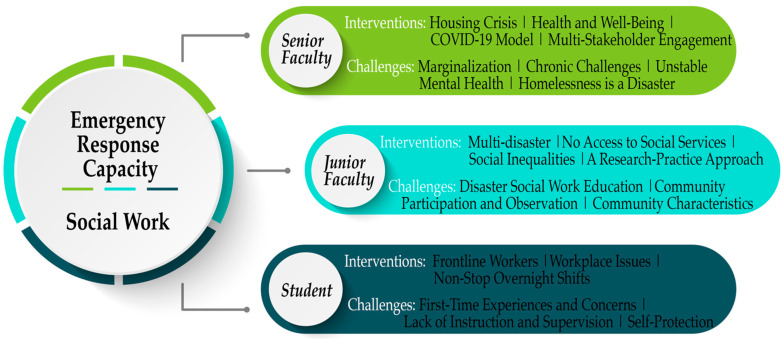
Data analysis structure. The three bars (senior faculty, junior faculty, and student) demonstrate the three authors’ critical reflection sub-themes that emerged in the data analysis regarding social work practice supporting PEHs. Under each sub-theme, bi-level sub-categories were developed to provide detailed supportive information, namely interventions and challenges. Different codes followed each subtheme.

The three authors shared their reflections independently during the meetings by email/phone communication. When one author presented his/her research and practice outcomes, the other two authors had opportunities to ask questions and critique this author’s reflections. Then, they collaboratively pooled their reflections to support the social work research and practice for PEHs during the pandemic. Furthermore, based on their educated experience, they comparatively consulted on what has already been taught in Canadian (Western) social work education, with the aim of improvement and advancement to effectively build social work practitioners’ coping capacity for disaster and emergency management, especially in reference to extreme events that have a worldwide impact, such as COVID-19 and climate change, with the hope that the Canadian knowledge could be mobilized to their international peers. The junior faculty (the first author) took meeting minutes at these meetings and emails/phone communications, primarily focusing on their critical reflection, critique, and comparison. These meeting minutes were shared with the other two authors for adding their observation notes, thus becoming the initial data in the conceptualization of this manuscript. The junior faculty applied interpretive and comparative lenses to analyze the three authors’ critical reflections through a thematic approach to identify social workers’ necessary emergency response efforts through effective interventions and COVID-19-driven challenges [53]. The data analysis structure and related codes and sub-themes (interventions and challenges) are presented in Figure 1.

### 3.3. Limitations

As shown in Table 1, the authors’ academic and demographic backgrounds unavoidably influence their field experience, critical reflections, observations, as well as data analysis and interpretation. These factors also impacted the first author (the junior faculty member) to take minutes and conduct data analysis. Social work education enabled the three authors to present their opinions from multiple standpoints and acknowledge their community-based social work experience with PEHs in the HRM. Accordingly, the junior faculty prepared this manuscript and completed the first draft based on thematic analysis outcomes. The other two authors reviewed the draft by reflecting on their previous and current social work interventions with PEHs and critically composing how the field-based practice, research, and learning experience underlie their cognitive social work capacity in Canada. Furthermore, the three authors’ critical reflections were HRM-based, which might not easily translate into the international context. Hence, the three authors collaboratively discussed the possibility of knowledge mobilization beyond the Canadian social work education landscape. The junior faculty finalized the manuscript based on the other authors’ suggestions. Major findings are presented below, reflecting the data analysis structure of Figure 1.

## 4. Results

Within different career stages, the three social workers (three authors) provided diverse interventions to support PEHs during COVID-19. Their challenges, associated with their social work interventions regarding policy, research, and practice, respectively, further illustrate, from their perspectives, areas for improvement in social work education and professional training.

### 4.1. The Senior Social Work Faculty: Advocating for Social Justice for PEHs

*Interventions*: As discussed above, the senior faculty’s research and practice have been primarily focused on PEHs: their entering into, surviving in, and for some, exiting from street life. As a co-founder of a local volunteer-based emergency homeless shelter for over 12 years, he has advocated for stable housing to support homeless persons’ health and well-being. During the current pandemic, this shelter developed the emergency response model, moving 25 guests (PEHs) into a hotel [35]. He has consistently promoted this emergency response model in the COVID-19 Working Group through daily virtual conferences with municipal and provincial governmental officials, public health professionals, and other stakeholders serving in the shelter systems. This cooperation built a virtual platform for different stakeholders, especially the related policy/decision makers, to share promising practices (e.g., other shelters’ strategies), update information and knowledge (e.g., the constantly changing public health requirements), advocate for better treatment (e.g., identify more available space), and explore emergency funding options.

*Challenges:* PEHs, living in extremely unsafe and precarious environments, are largely excluded from civil society and rarely receive the same opportunities as “normal” citizens. Based on the more than 12-year engagement with PEHs, the senior faculty argued that PEH’s marginalization status has been getting worse. The COVID-19 core public health protocols primarily neglected them during the first wave of emergency response (e.g., stay at home and self-isolation) because they did not have the basic living right of staying home, as they did not have a home to stay in. Additionally, PEHs usually present chronic challenges, such as struggles with substance use, and unregulated levels of trauma, and family violence. Due to the COVID-19-triggered closures of non-essential social services, these needs went unaddressed. Furthermore, the pandemic triggered tremendous uncertainties, further aggravated by fear, stress, and dread, worsening PEHs’ unstable mental health status, calling for social workers’ and other mental health professionals’ interventions [35]. Reflecting on these systemic inequalities that PEHs experience on a daily basis, a larger context of simply “not knowing” associated with the unpredictable nature of COVID-19 has encouraged social workers to convince governmental partners and policy decision-makers to deeply examine the pandemic-triggered homelessness crisis through the lens of public health and social justice [43]. While homelessness was seen as a disaster, in and of itself before the pandemic, COVID-19 articulated and amplified root injustices that stimulated the social worker’s ongoing efforts for PEH advocacy, requesting the community of providers to re-examine all levels of essential support.

### 4.2. The Junior Social Work Faculty: Integrating Disaster Research Components into Pedagogy

*Interventions*: During the academic years 2019–2020 and 2020–2021, as a disaster researcher and practitioner, this junior faculty member taught social work research methods courses at the undergraduate and graduate levels. During this period, HRM was devastated by several critical extreme events, including COVID-19, Hurricane Dorian, and a mass shooting [54]. Undoubtedly, PEHs were among the ones to suffer the greatest. In particular, the temporary closure of homeless shelters due to Hurricane Dorian and COVID-19 disabled PEHs’ access to basic living services [35,54]. This societal background has built a living platform that encouraged him to integrate disaster-specific components into current social work pedagogies. He contextualized his disaster research and practice expertise within the local community and requested his students to identify the root causes of homelessness through community-based observation and comparison of the PEHs’ inequalities before and during COVID-19 and other disaster events. This approach has stimulated the students’ research interests and has informed their practice strategies to move beyond disaster settings. More importantly, this approach has been found to empower the students to consider and develop holistic social work interventions that address the fundamental inequalities that contribute to homelessness.

*Challenges*: Since professional social work education continues to lack curriculum and training that reflects the complexities associated with humanitarian services in the hazards and disaster fields [55], when introduced to disaster-specific components, most social work students were unsure how social work research and practice directly contribute toward ameliorating extreme events. The instructor guided the students, for example, to utilize community-based participation and observation approaches to understand more deeply how disaster-specific catastrophic influences compounded with the ongoing challenges that PEHs continually encountered pre-disaster, further worsened their vulnerabilities and placed them in an arduous post-disaster recovery process. Accordingly, students were then able to identify that social work knowledge and skills could advocate for PEHs and other vulnerable people’s rights, provide crisis interventions (e.g., mental health support), and map community-based resources that would fulfill their basic living requirements (e.g., access to food and accommodation) and address their particular needs. However, most social work education and special training have not effectively connected and conceptualized social work interventions in community-based, disaster, and non-disaster scenarios, to address fundamental issues of diverse inequalities. Community-targeted social work research could strengthen the students’ disaster-driven capacities by helping them to more comprehensively understand the community’s unique characteristics, such as special needs for different demographic groups and different types of community-based resources, to better advocate for vulnerable and marginalized inhabitants.

### 4.3. The Undergraduate Social Work Student: Pursuing Disaster-Specific Social Work Training

*Interventions:* The “topsy-turvy” world triggered by COVID-19 gave rise to new emergency pop-up shelters in Halifax, where this young social worker practiced as a frontline shelter worker for the first time in her life. The pop-up shelters were an innovative crisis response approach engendered by the pandemic; they were organized and staffed primarily by individuals unfamiliar with social work and homelessness. “There’s no training” was her first impression of stepping into the shelter system. There was no supervision, no structure, few tasks, few guests (PEHs), and a massive binder with instructions and policies associated with public health instructions. After nearly a month off work, she was sent to a new workplace due to a non-coronavirus-caused illness. The next day, she was transferred to work at one of the hotel locations, once the hotel model was in effect. Later, she discovered that the shelter moved over 30 guests to the hotel isolation system because a positive COVID-19 case was identified at one of the pop-up shelters. As with the previous location, there were no instructions, no supervisors, and a lack of personal protective equipment (PPE). Her co-workers also had no clue what was happening until shelter guests started trickling into the hotel lobby. During her long and uncertain shift (e.g., 17-h shift), she had to figure out what to do almost entirely on her own. This on-the-spot learning process built her capacity and stimulated her to re-examine the social work education and training she received at the university level.

*Challenges*: When she called for support because some guests (PEHs) were confused and overwhelmed (which led to acting out), she was told to report directly to the police. Her social work expertise recognized that involving the police was unrealistic and inappropriate. It would put guests at risk, cause them to feel unsafe, and not help the situation at all. Although she wished she could cope with this complex situation as an experienced social worker, the COVID-19 pandemic brought about so many “first-time” experiences and concerns. Social work professional ethics required her to focus on clients’ realities; however, having not received in-shelter training, having limited disaster and emergency management related social work knowledge and skills, and the challenges she faced in swiftly obtaining the knowledge and skills needed, converged to reduce her emergency response capacity. She tried her best to manage some guests who were going through forced withdrawal due to the unexpected quarantine, some who were experiencing the risk of overdosing, some who were threatening suicide, some who were setting their room on fire, and some who were harassing and threatening her, even after being banned from the premises. All this happened during these non-stop overnight shifts; she found that there was no chance for her to breathe. Since basic staff support was almost impossible in the temporary shelters, self-care and self-maintenance were vital for the workers’ health and overall well-being. This student’s challenges motivated the two social work faculty members to explore the roles of in-class and field social work education, in preparing future social workers for the incoming waves of COVID-19 and other potential extreme events. Traditional social work education establishes the students’ practice foundation, while self-care is a sustainable way to enable young social workers to continually obtain experience from the field and adapt field knowledge and skills to different disaster contexts.

## 5. Discussion

The COVID-19 pandemic enables international discussion regarding social workers’ roles, not only in social services and public health interventions, but also as advocates for the rights of vulnerable people who are inextricably linked to societal influences (e.g., economic, social, cultural, and political) [56]. The three social workers’ COVID-19-driven interventions and challenges in supporting PEHs illustrate the following three types of social work effort rooted in different career stages, which contribute to building social work-specific emergency response capacity.

### 5.1. The Senior Faculty: Top-Down Advocacy Effort of Social Development and Policy

COVID-19 has re-articulated how fragile our capitalist, neoliberal ways of organizing society are [57] and has re-awakened debates surrounding essential employment, precarious employment, health and work, societal safety structures, and the profound discrepancy within our communities, especially regarding ethnicity, gender, class, geography, and age [58,59]. The coronavirus-driven social and economic challenges urgently call for the political support of social service agencies, especially homeless shelters, which have proven to be underfunded and have not received enough public attention either domestically or internationally. Although everyone has differently experienced the weight of this pandemic, it has become clear that factors such as race, culture, gender, social and economic status, age, health, and geography have all compounded to impact individuals’ health and well-being [60,61]. The COVID-19 pandemic has again roused awareness to strengthen the core principles of social justice and anti-oppressive policy and practice, and has propelled social workers to advocate for social justice in the context of compound vulnerabilities to advance community social development.

Social workers coordinate disaster relief, advocate for vulnerable people’s rights, and improve access to government-supported and/or community-based services and resources [3]. The daily meetings of the COVID-19 working group for PEHs that the senior faculty attended formed a virtual platform for policy decision-makers and government stakeholders to hear the frontline responders’ voices in planning relief activities. This enabled the government stakeholders to prepare for potential challenges and improve the relevant policies. In other words, the communication channels between the ground level and policymakers allowed for strategic and intentional service delivery mobilization, to better serve the PEHs who had been disproportionately affected by COVID-19 and more generally, for other vulnerable and marginalized groups who have also been lethally hit by current disasters and who will be in the future. This communication platform initiated the first step of social work interventions, empowering the grassroots voices of frontline workers in the decision-making process [62,63]. Then, social workers continually advocate for the development of and/or enhancement of relevant policy and/or community-based outreach, which should engage the grassroots voices of the PEHs, to create a greater fundamental understanding of their unique needs, so that appropriate interventions could be customized. Namely, social workers empower grassroots agencies in the decision-making process and advocate for “top-down” policy support to address the root causes of homelessness and other social inequalities. Hence, this platform illustrates an effective approach for the international communities to collaboratively develop research, practice, and policy interventions to support PEHs and other vulnerable and marginalized groups. The three social workers’ engagement in this process has provided valuable references for their international social work peers to promote PEH advocacy.

### 5.2. The Junior Faculty: Bottom-Up Cognitive Effort of Comprehending Communities’ Dynamics

A nuanced understanding of local communities’ diversities and resources would primarily advance the frontline social workers’ community mapping interventions, enabling them to swiftly bridge clients (PEHs in this article) with appropriate resources and services in disaster settings [64]. Hence, building community-driven research capacity could assist the community-based social workers to better appreciate their community dynamics and identify their “community assets”, equipping them to support the local dwellers’ demands facing any crises, especially for dwellers within the vulnerable and marginalized groups, such as PEHs. Pedagogical approaches should be contextualized within a given local community’s parameters through engaging community-based learning approaches (e.g., surveys, case studies, and participant observations) to enhance students’ community-based knowledge and skills. Locally-specific social work interventions are the most welcomed by a community’s residents and stimulate collaboration among different stakeholders [65]. Advancing the students’ understanding of their communities enables them to develop community-driven strategies that effectively serve the local community in both disaster and non-disaster settings. Adopting community-based research from an anti-oppressive practice and social justice standpoint has been included in general social work training [66]. To a great extent, disaster can instigate various societal inequalities that create lived environments, stimulating students to utilize these bottom-up skills to address existing and emerging issues within the particular circumstance of a given disaster situation [67].

In the hazards and disaster research field, the U.S. National Science Foundation-funded CONVERGE hazards and disaster research initiatives are designing essential training modules to accelerate the education for the next generation of hazards and disaster researchers [68]. These training modules, such as *Social Vulnerability and Disasters* and *Cultural Competence in Hazards and Disaster Research*, assist hazards and disaster researchers in their community-based, scientifically rigorous research and ethical practices [69]. Since social work educational programs worldwide have already begun to address the social, cultural, and related societal vulnerabilities [70,71,72], these training modules, propelled by the global disaster context, encourage professional social work education to adopt and/or develop their disaster-specific educational modules in support of these efforts, by stabilizing potential procedures, policies, and educational structures. The global COVID-19 outbreak that has initiated the development of community-driven disaster response strategies urgently calls for these modules for social work education.

### 5.3. The Student: Disaster-Specific Effort of Self-Protection and Self-Care

The COVID-19 pandemic, which has uncovered the weakness of emergency response strategies at different levels, has stimulated community-based service agencies to rethink their emergency management strategies [10]. This will generate valuable field education and on-site training opportunities for the next generation of practitioners. Qualified and experienced social work practitioners must be engaged to promote this process. A growing concern among early career social work practitioners, as indicated in the young social work practitioner’s case above, has been whether tangible long-term planning will become part of these emergency response strategies, so that the safety, health, and overall well-being of frontline workers will be recognized as a requirement. The ongoing trend of treating COVID-19-related social services as ‘temporary’ and, in so doing, considering them undeserving of the relevant organization, training, and financial support, leaves the safety of practitioners, clients, and the general public at greater risk of contracting COVID-19, and of the deterioration of their overall health and well-being. This improved, ongoing strategic planning not only promotes the quality of agencies’ holistic social service, better serving the affected communities, but also significantly contributes to a sustainable model of staff protection during this pandemic and beyond, further strengthening agencies’ ongoing development.

Although self-care is vital for social work education [3], working on the frontline of COVID-19 exposes social workers to extra burdens, including physical health risks and psychological impacts. Yanay and Benjamin [11] argue that a deep, sympathetic understanding of local inhabitants’ needs urges social workers to take on responsibilities beyond their capacities, further worsening their pressure, stress, and anxiety. The self-care components in the current social work educational model need to encompass the compounded influences of extreme events, guaranteeing that social workers be provided with support for their health and well-being while giving high-quality service to their clients [73]. Furthermore, “debriefing social workers and learning about their individual experiences is both helpful and healing, but it is also essential for building a knowledge base for future cases” [11] (p. 273). As an example, during the orientation of the 2020–2021 academic year for the School of Social Work at Dalhousie University, the two faculty members facilitated a debriefing section for students to freely share their COVID-19-related experiences, challenges, concerns, and promising practice. Included as an essential social work education component, this roundtable discussion was highly welcomed by students. Other educational organizations worldwide could implement a similar check-in section for recent graduates and frontline service agencies, enabling their staff to share their frontline experiences, address their practice challenges, and give impetus for receiving support for their mental health and overall well-being.

## 6. Conclusions

The global context of the increasing numbers of extreme events has further confirmed the rapid growth and dire need for disaster-related social work practice domestically and internationally. The complexity of extreme events potentially increases the challenges of social work emergency response efforts, especially for vulnerable and marginalized populations. Utilizing these three social workers’ COVID-19-driven research and practice within the homeless sector in HRM as an example, this article presents their critical reflections on interventions and challenges in alignment with their career stages. Their COVID-19-specific experience opens a window for advancing social workers’ threefold emergency response components: top-down advocacy effort of social development and policy, bottom-up cognitive effort of comprehending communities’ dynamics, and disaster-specific self-care capacity. These three components demonstrate the ongoing and increasing need for social work education to further build the comprehensive disaster-specific capability for the next generation of practitioners in Canada and internationally.

The interconnections among social work research, education, and practice strengthen the disaster-oriented components in social work education and provide disaster-driven practice supervision, to prepare emerging professionals for diverse clients, emerging research, and supportive policy development. Critical reflection on experiential knowledge and evidence-based strategies are crucial for emergency response personnel to learn from the current global pandemic and prepare them for potential extreme events. The senior and junior faculty’s experience encourages social work professionals to develop top-down policy support and bottom-up community strategies in disaster settings. The student’s endeavors highlight the necessity of strengthening disaster-specific self-maintenance. Merging these social workers’ experiences calls for related in-classroom pedagogical enhancement and on-site field education training, applied to the Canadian context in particular and the international landscape in general. Stimulating ongoing dialogue among social work researchers, educators, and practitioners will wholly incorporate their experiences into any new educational materials and even policy shifts. Future research could elaborate on this aspect, supporting PEHs and other vulnerable and marginalized groups in the diverse global context of extreme events.

## Figures and Tables

**Table 1:** Team’s academic and demographic information.

	Career Stage	Senior Faculty	Junior Faculty	Student
**Research, training, practice experience**	Disaster and emergency management	No	Yes	No
	PEHs	Yes	No	No
**Demographic variables**	Gender and sexual minority	No	Yes	Yes
	Immigration status (culture)	No	Yes	No
	Ethnic minority	No	Yes	No

## Data Availability

Not applicable.
